# *N*′-[(Biphenyl-4-yl)methyl­ene]-2-[(3,5-di-*tert*-butyl-4-hydroxy­benz­yl)sulfan­yl]acetohydrazide

**DOI:** 10.1107/S1600536810006884

**Published:** 2010-03-03

**Authors:** Wagee A. Yehye, Azhar Ariffin, Noorsaadah Abdul Rahman, Seik Weng Ng

**Affiliations:** a Department of Chemistry, University of Malaya, 50603 Kuala Lumpur, Malaysia

## Abstract

In the title compound, C_30_H_36_N_2_O_2_S, the dihedral angle between the two aromatic rings of the biphenyl residue is 31.2 (1)°. The two methyl­ene C atoms subtend an angle of 99.9 (1)° at the S atom. In the crystal, mol­ecules form inversion dimers linked by pairs of N—H⋯O hydrogen bonds. The hydroxyl group is shielded by the *tert*-butyl residues and is therefore not involved in any hydrogen bonding.

## Related literature

When heated in acidified ethanol the compound gave biphenyl-4-carbaldehyde azine; see: Yehye *et al.* (2008 ▶).
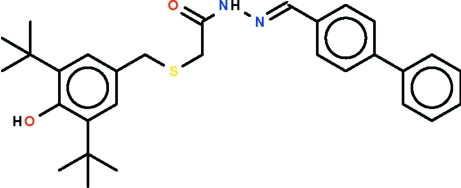


## Experimental

### 

#### Crystal data

C_30_H_36_N_2_O_2_S
*M* *_r_* = 488.67
Triclinic, 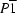

*a* = 9.1104 (11) Å
*b* = 10.5601 (12) Å
*c* = 15.5146 (18) Å
α = 104.435 (2)°
β = 102.805 (2)°
γ = 97.559 (2)°
*V* = 1381.7 (3) Å^3^
*Z* = 2
Mo *K*α radiation
μ = 0.15 mm^−1^
*T* = 293 K
0.40 × 0.10 × 0.10 mm

#### Data collection

Bruker SMART APEX diffractometer
Absorption correction: multi-scan (*SADABS*; Sheldrick, 1996 ▶) *T*_min_ = 0.944, *T*_max_ = 0.986
10977 measured reflections
4873 independent reflections
3404 reflections with *I* > 2σ(*I*)
*R*_int_ = 0.035

#### Refinement

*R*[*F* ^2^ > 2σ(*F* ^2^)] = 0.047
*wR*(*F* ^2^) = 0.135
*S* = 1.04
4873 reflections
324 parameters
2 restraints
H atoms treated by a mixture of independent and constrained refinement
Δρ_max_ = 0.17 e Å^−3^
Δρ_min_ = −0.19 e Å^−3^

### 

Data collection: *APEX2* (Bruker, 2009 ▶); cell refinement: *SAINT* (Bruker, 2009 ▶); data reduction: *SAINT*; program(s) used to solve structure: *SHELXS97* (Sheldrick, 2008 ▶); program(s) used to refine structure: *SHELXL97* (Sheldrick, 2008 ▶); molecular graphics: *X-SEED* (Barbour, 2001 ▶); software used to prepare material for publication: *publCIF* (Westrip, 2010 ▶).

## Supplementary Material

Crystal structure: contains datablocks global, I. DOI: 10.1107/S1600536810006884/bt5202sup1.cif

Structure factors: contains datablocks I. DOI: 10.1107/S1600536810006884/bt5202Isup2.hkl

Additional supplementary materials:  crystallographic information; 3D view; checkCIF report

## Figures and Tables

**Table 1:** Hydrogen-bond geometry (Å, °)

*D*—H⋯*A*	*D*—H	H⋯*A*	*D*⋯*A*	*D*—H⋯*A*
N1—H1⋯O2^i^	0.87 (1)	1.97 (1)	2.827 (2)	174 (2)

Symmetry code: (i) 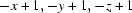.

**Fig. 1.**
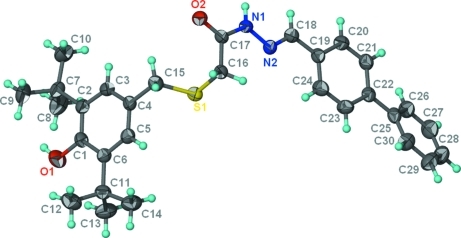
Anisotropic displacement ellipsoid plot (Barbour, 2001) of C30H36N2O2S at the the 50% probability level; hydrogen atoms are drawn as spheres of arbitrary radius.

## supplementary crystallographic information

### Experimental

2-(3,5-Di-*tert*-butyl-4-hydroxybenzylthio)acetohydrazine (0.5 g, 1.54 mmol) and 4-phenylbenzaldehyde (0.28 g, 1.54 mmol) were stirred in ethanol (10 ml) for 2 h. The resulting solid was collected and recrystallized from ethanol to give the Schiff base as large prismatic crystals in 90% yield. The formulation was assumed from ^1^H NMR spectral analysis.

### Refinement

Carbon-bound H-atoms were placed in calculated positions (C—H 0.93 to 0.98 Å) and were included in the refinement in the riding model approximation, with *U*(H) set to 1.2 to 1.5*U*(C). The hydroxy and amino H-atoms were located in a difference Fourier map, and were refined isotropically with distance restraints of O–H 0.84±0.01 Å and N–H 0.86±0.01 Å.

### Crystal data

**Table:**

C_30_H_36_N_2_O_2_S	*Z* = 2
*M**_r_* = 488.67	*F*(000) = 524
Triclinic, *P*1	*D*_x_ = 1.175 Mg m^−^^3^
Hall symbol: -P 1	Mo *K*α radiation, λ = 0.71073 Å
*a* = 9.1104 (11) Å	Cell parameters from 2001 reflections
*b* = 10.5601 (12) Å	θ = 2.3–21.2°
*c* = 15.5146 (18) Å	µ = 0.14 mm^−^^1^
α = 104.435 (2)°	*T* = 293 K
β = 102.805 (2)°	Prism, colorless
γ = 97.559 (2)°	0.40 × 0.10 × 0.10 mm
*V* = 1381.7 (3) Å^3^	

### Data collection

**Table:**

Bruker SMART APEX diffractometer	4873 independent reflections
Radiation source: fine-focus sealed tube	3404 reflections with *I* > 2σ(*I*)
graphite	*R*_int_ = 0.035
ω scans	θ_max_ = 25.0°, θ_min_ = 2.0°
Absorption correction: multi-scan (*SADABS*; Sheldrick, 1996)	*h* = −10→10
*T*_min_ = 0.944, *T*_max_ = 0.986	*k* = −12→12
10977 measured reflections	*l* = −16→18

### Refinement

**Table:**

Refinement on *F*^2^	Primary atom site location: structure-invariant direct methods
Least-squares matrix: full	Secondary atom site location: difference Fourier map
*R*[*F*^2^ > 2σ(*F*^2^)] = 0.047	Hydrogen site location: inferred from neighbouring sites
*wR*(*F*^2^) = 0.135	H atoms treated by a mixture of independent and constrained refinement
*S* = 1.04	*w* = 1/[σ^2^(*F*_o_^2^) + (0.0664*P*)^2^ + 0.0264*P*] where *P* = (*F*_o_^2^ + 2*F*_c_^2^)/3
4873 reflections	(Δ/σ)_max_ = 0.001
324 parameters	Δρ_max_ = 0.17 e Å^−^^3^
2 restraints	Δρ_min_ = −0.19 e Å^−^^3^

### Fractional atomic coordinates and isotropic or equivalent isotropic displacement parameters (Å^2^)

**Table:**

	*x*	*y*	*z*	*U*_iso_*/*U*_eq_	
S1	0.76556 (7)	0.68306 (6)	0.78801 (4)	0.0490 (2)	
O1	0.5035 (2)	0.7947 (2)	1.15128 (12)	0.0773 (6)	
H1O	0.414 (2)	0.758 (5)	1.146 (4)	0.20 (2)*	
O2	0.54723 (17)	0.65943 (15)	0.58105 (10)	0.0543 (4)	
N1	0.7059 (2)	0.51789 (18)	0.55439 (12)	0.0445 (5)	
H1	0.6318 (18)	0.4645 (17)	0.5098 (11)	0.048 (6)*	
N2	0.8470 (2)	0.48554 (18)	0.57906 (12)	0.0449 (4)	
C1	0.5274 (2)	0.7853 (2)	1.06541 (15)	0.0472 (6)	
C2	0.4146 (2)	0.7092 (2)	0.98530 (15)	0.0443 (5)	
C3	0.4476 (2)	0.7061 (2)	0.90205 (15)	0.0475 (6)	
H3	0.3756	0.6556	0.8477	0.057*	
C4	0.5832 (2)	0.7749 (2)	0.89644 (15)	0.0465 (6)	
C5	0.6914 (2)	0.8472 (2)	0.97723 (15)	0.0468 (6)	
H5	0.7831	0.8931	0.9736	0.056*	
C6	0.6687 (2)	0.8541 (2)	1.06363 (15)	0.0428 (5)	
C7	0.2608 (2)	0.6336 (2)	0.98892 (17)	0.0538 (6)	
C8	0.2880 (3)	0.5279 (3)	1.0406 (2)	0.0774 (8)	
H8A	0.3423	0.4667	1.0100	0.116*	
H8B	0.1910	0.4801	1.0409	0.116*	
H8C	0.3477	0.5711	1.1029	0.116*	
C9	0.1710 (3)	0.7315 (3)	1.0355 (2)	0.0724 (8)	
H9A	0.0774	0.6826	1.0394	0.109*	
H9B	0.1475	0.7919	0.9997	0.109*	
H9C	0.2323	0.7811	1.0964	0.109*	
C10	0.1580 (3)	0.5607 (3)	0.8920 (2)	0.0779 (8)	
H10A	0.2075	0.4953	0.8612	0.117*	
H10B	0.1405	0.6238	0.8575	0.117*	
H10C	0.0616	0.5174	0.8963	0.117*	
C11	0.7924 (3)	0.9341 (2)	1.15209 (15)	0.0513 (6)	
C12	0.7331 (3)	1.0494 (3)	1.20689 (19)	0.0837 (9)	
H12A	0.6451	1.0141	1.2243	0.126*	
H12B	0.7047	1.1066	1.1693	0.126*	
H12C	0.8124	1.0995	1.2613	0.126*	
C13	0.8421 (3)	0.8430 (3)	1.21158 (19)	0.0748 (8)	
H13A	0.7556	0.8054	1.2292	0.112*	
H13B	0.9211	0.8940	1.2659	0.112*	
H13C	0.8808	0.7725	1.1768	0.112*	
C14	0.9374 (3)	0.9949 (3)	1.13007 (19)	0.0720 (8)	
H14A	1.0121	1.0454	1.1865	0.108*	
H14B	0.9115	1.0525	1.0924	0.108*	
H14C	0.9793	0.9248	1.0976	0.108*	
C15	0.6138 (3)	0.7729 (3)	0.80454 (16)	0.0578 (6)	
H15A	0.6433	0.8635	0.8025	0.069*	
H15B	0.5212	0.7302	0.7553	0.069*	
C16	0.7967 (2)	0.7190 (2)	0.68495 (14)	0.0445 (5)	
H16A	0.7939	0.8118	0.6893	0.053*	
H16B	0.8969	0.7036	0.6786	0.053*	
C17	0.6743 (2)	0.6309 (2)	0.60228 (14)	0.0415 (5)	
C18	0.8649 (3)	0.3747 (2)	0.53133 (15)	0.0486 (6)	
H18	0.7843	0.3207	0.4826	0.058*	
C19	1.0115 (2)	0.3318 (2)	0.55261 (15)	0.0448 (5)	
C20	1.0305 (3)	0.2105 (2)	0.50071 (16)	0.0520 (6)	
H20	0.9502	0.1578	0.4513	0.062*	
C21	1.1672 (3)	0.1671 (2)	0.52141 (16)	0.0508 (6)	
H21	1.1770	0.0852	0.4857	0.061*	
C22	1.2902 (2)	0.2427 (2)	0.59426 (15)	0.0438 (5)	
C23	1.2704 (3)	0.3650 (2)	0.64509 (16)	0.0539 (6)	
H23	1.3511	0.4186	0.6939	0.065*	
C24	1.1346 (3)	0.4084 (2)	0.62486 (16)	0.0549 (6)	
H24	1.1251	0.4906	0.6602	0.066*	
C25	1.4349 (3)	0.1944 (2)	0.61918 (15)	0.0470 (6)	
C26	1.4334 (3)	0.0584 (3)	0.60401 (17)	0.0578 (6)	
H26	1.3410	−0.0024	0.5761	0.069*	
C27	1.5670 (3)	0.0121 (3)	0.62980 (19)	0.0703 (8)	
H27	1.5640	−0.0788	0.6199	0.084*	
C28	1.7033 (3)	0.1010 (4)	0.6700 (2)	0.0748 (8)	
H28	1.7932	0.0703	0.6875	0.090*	
C29	1.7081 (3)	0.2344 (3)	0.68445 (19)	0.0719 (8)	
H29	1.8015	0.2940	0.7112	0.086*	
C30	1.5748 (3)	0.2822 (3)	0.65969 (18)	0.0607 (7)	
H30	1.5794	0.3734	0.6703	0.073*	

### Atomic displacement parameters (Å^2^)

**Table:**

	*U*^11^	*U*^22^	*U*^33^	*U*^12^	*U*^13^	*U*^23^
S1	0.0509 (4)	0.0565 (4)	0.0402 (3)	0.0122 (3)	0.0105 (3)	0.0156 (3)
O1	0.0721 (13)	0.1141 (16)	0.0468 (11)	−0.0004 (12)	0.0283 (10)	0.0237 (10)
O2	0.0481 (9)	0.0556 (10)	0.0505 (10)	0.0197 (8)	0.0053 (7)	0.0020 (7)
N1	0.0409 (11)	0.0479 (11)	0.0375 (11)	0.0131 (9)	0.0044 (9)	0.0024 (9)
N2	0.0435 (11)	0.0508 (11)	0.0427 (11)	0.0165 (9)	0.0120 (8)	0.0135 (9)
C1	0.0484 (13)	0.0578 (14)	0.0400 (13)	0.0093 (11)	0.0186 (11)	0.0169 (11)
C2	0.0395 (12)	0.0499 (13)	0.0463 (13)	0.0104 (10)	0.0133 (10)	0.0166 (11)
C3	0.0421 (12)	0.0549 (14)	0.0426 (13)	0.0100 (10)	0.0075 (10)	0.0119 (11)
C4	0.0441 (13)	0.0619 (15)	0.0389 (13)	0.0151 (11)	0.0156 (10)	0.0177 (11)
C5	0.0415 (12)	0.0554 (14)	0.0473 (14)	0.0050 (10)	0.0160 (11)	0.0203 (11)
C6	0.0415 (12)	0.0453 (13)	0.0432 (13)	0.0070 (10)	0.0119 (10)	0.0158 (10)
C7	0.0394 (12)	0.0612 (15)	0.0635 (16)	0.0065 (11)	0.0167 (11)	0.0220 (13)
C8	0.0623 (17)	0.0742 (19)	0.111 (2)	0.0098 (14)	0.0326 (17)	0.0471 (18)
C9	0.0513 (15)	0.086 (2)	0.090 (2)	0.0194 (14)	0.0322 (15)	0.0283 (16)
C10	0.0483 (16)	0.086 (2)	0.084 (2)	−0.0105 (14)	0.0134 (14)	0.0132 (16)
C11	0.0503 (13)	0.0509 (14)	0.0456 (13)	0.0032 (11)	0.0079 (11)	0.0093 (11)
C12	0.092 (2)	0.077 (2)	0.0620 (18)	0.0160 (17)	0.0096 (16)	−0.0065 (15)
C13	0.0641 (17)	0.088 (2)	0.0652 (18)	0.0056 (15)	−0.0033 (14)	0.0320 (16)
C14	0.0584 (16)	0.0727 (18)	0.0679 (18)	−0.0148 (14)	0.0045 (13)	0.0154 (14)
C15	0.0559 (15)	0.0806 (18)	0.0440 (14)	0.0211 (13)	0.0167 (11)	0.0238 (13)
C16	0.0409 (12)	0.0466 (13)	0.0419 (13)	0.0035 (10)	0.0127 (10)	0.0067 (10)
C17	0.0439 (13)	0.0447 (13)	0.0350 (12)	0.0099 (10)	0.0108 (10)	0.0094 (10)
C18	0.0481 (13)	0.0527 (14)	0.0438 (13)	0.0136 (11)	0.0105 (11)	0.0111 (11)
C19	0.0491 (13)	0.0489 (13)	0.0411 (13)	0.0158 (11)	0.0152 (11)	0.0151 (11)
C20	0.0504 (14)	0.0530 (14)	0.0471 (14)	0.0117 (11)	0.0086 (11)	0.0076 (11)
C21	0.0560 (15)	0.0455 (13)	0.0521 (14)	0.0184 (11)	0.0175 (12)	0.0092 (11)
C22	0.0487 (13)	0.0494 (14)	0.0409 (12)	0.0140 (10)	0.0167 (10)	0.0202 (11)
C23	0.0533 (14)	0.0534 (15)	0.0484 (14)	0.0148 (12)	0.0020 (11)	0.0108 (12)
C24	0.0650 (16)	0.0494 (14)	0.0490 (14)	0.0222 (12)	0.0106 (12)	0.0102 (11)
C25	0.0527 (14)	0.0565 (15)	0.0424 (13)	0.0185 (11)	0.0204 (11)	0.0223 (11)
C26	0.0605 (16)	0.0606 (16)	0.0577 (16)	0.0232 (12)	0.0166 (12)	0.0200 (12)
C27	0.080 (2)	0.0734 (19)	0.0688 (18)	0.0430 (17)	0.0213 (16)	0.0245 (15)
C28	0.0611 (18)	0.106 (3)	0.0719 (19)	0.0418 (18)	0.0209 (15)	0.0365 (18)
C29	0.0492 (15)	0.096 (2)	0.076 (2)	0.0150 (15)	0.0146 (14)	0.0374 (17)
C30	0.0555 (15)	0.0686 (17)	0.0667 (17)	0.0133 (13)	0.0179 (13)	0.0326 (14)

### Geometric parameters (Å, °)

**Table:**

S1—C16	1.804 (2)	C12—H12C	0.9600
S1—C15	1.805 (2)	C13—H13A	0.9600
O1—C1	1.379 (3)	C13—H13B	0.9600
O1—H1O	0.83 (1)	C13—H13C	0.9600
O2—C17	1.230 (2)	C14—H14A	0.9600
N1—C17	1.342 (3)	C14—H14B	0.9600
N1—N2	1.371 (2)	C14—H14C	0.9600
N1—H1	0.87 (1)	C15—H15A	0.9700
N2—C18	1.271 (3)	C15—H15B	0.9700
C1—C2	1.402 (3)	C16—C17	1.500 (3)
C1—C6	1.402 (3)	C16—H16A	0.9700
C2—C3	1.383 (3)	C16—H16B	0.9700
C2—C7	1.539 (3)	C18—C19	1.462 (3)
C3—C4	1.380 (3)	C18—H18	0.9300
C3—H3	0.9300	C19—C24	1.386 (3)
C4—C5	1.379 (3)	C19—C20	1.387 (3)
C4—C15	1.508 (3)	C20—C21	1.383 (3)
C5—C6	1.387 (3)	C20—H20	0.9300
C5—H5	0.9300	C21—C22	1.387 (3)
C6—C11	1.534 (3)	C21—H21	0.9300
C7—C10	1.530 (3)	C22—C23	1.392 (3)
C7—C9	1.540 (3)	C22—C25	1.482 (3)
C7—C8	1.543 (3)	C23—C24	1.375 (3)
C8—H8A	0.9600	C23—H23	0.9300
C8—H8B	0.9600	C24—H24	0.9300
C8—H8C	0.9600	C25—C30	1.387 (3)
C9—H9A	0.9600	C25—C26	1.394 (3)
C9—H9B	0.9600	C26—C27	1.385 (3)
C9—H9C	0.9600	C26—H26	0.9300
C10—H10A	0.9600	C27—C28	1.367 (4)
C10—H10B	0.9600	C27—H27	0.9300
C10—H10C	0.9600	C28—C29	1.363 (4)
C11—C12	1.535 (3)	C28—H28	0.9300
C11—C14	1.536 (3)	C29—C30	1.388 (3)
C11—C13	1.535 (3)	C29—H29	0.9300
C12—H12A	0.9600	C30—H30	0.9300
C12—H12B	0.9600		
			
C16—S1—C15	99.92 (10)	C11—C13—H13C	109.5
C1—O1—H1O	110 (4)	H13A—C13—H13C	109.5
C17—N1—N2	120.93 (18)	H13B—C13—H13C	109.5
C17—N1—H1	117.2 (14)	C11—C14—H14A	109.5
N2—N1—H1	121.8 (14)	C11—C14—H14B	109.5
C18—N2—N1	116.47 (18)	H14A—C14—H14B	109.5
O1—C1—C2	120.5 (2)	C11—C14—H14C	109.5
O1—C1—C6	116.5 (2)	H14A—C14—H14C	109.5
C2—C1—C6	122.98 (19)	H14B—C14—H14C	109.5
C3—C2—C1	116.67 (19)	C4—C15—S1	109.84 (15)
C3—C2—C7	121.2 (2)	C4—C15—H15A	109.7
C1—C2—C7	122.10 (19)	S1—C15—H15A	109.7
C4—C3—C2	122.6 (2)	C4—C15—H15B	109.7
C4—C3—H3	118.7	S1—C15—H15B	109.7
C2—C3—H3	118.7	H15A—C15—H15B	108.2
C5—C4—C3	118.6 (2)	C17—C16—S1	109.61 (14)
C5—C4—C15	120.1 (2)	C17—C16—H16A	109.7
C3—C4—C15	121.4 (2)	S1—C16—H16A	109.7
C4—C5—C6	122.6 (2)	C17—C16—H16B	109.7
C4—C5—H5	118.7	S1—C16—H16B	109.7
C6—C5—H5	118.7	H16A—C16—H16B	108.2
C5—C6—C1	116.5 (2)	O2—C17—N1	121.11 (19)
C5—C6—C11	121.18 (19)	O2—C17—C16	120.80 (19)
C1—C6—C11	122.28 (19)	N1—C17—C16	118.04 (19)
C10—C7—C9	106.5 (2)	N2—C18—C19	120.3 (2)
C10—C7—C2	111.37 (19)	N2—C18—H18	119.8
C9—C7—C2	110.51 (19)	C19—C18—H18	119.8
C10—C7—C8	107.4 (2)	C24—C19—C20	117.8 (2)
C9—C7—C8	110.5 (2)	C24—C19—C18	121.9 (2)
C2—C7—C8	110.45 (19)	C20—C19—C18	120.2 (2)
C7—C8—H8A	109.5	C21—C20—C19	120.9 (2)
C7—C8—H8B	109.5	C21—C20—H20	119.5
H8A—C8—H8B	109.5	C19—C20—H20	119.5
C7—C8—H8C	109.5	C20—C21—C22	121.5 (2)
H8A—C8—H8C	109.5	C20—C21—H21	119.3
H8B—C8—H8C	109.5	C22—C21—H21	119.3
C7—C9—H9A	109.5	C21—C22—C23	117.1 (2)
C7—C9—H9B	109.5	C21—C22—C25	121.8 (2)
H9A—C9—H9B	109.5	C23—C22—C25	121.1 (2)
C7—C9—H9C	109.5	C24—C23—C22	121.6 (2)
H9A—C9—H9C	109.5	C24—C23—H23	119.2
H9B—C9—H9C	109.5	C22—C23—H23	119.2
C7—C10—H10A	109.5	C23—C24—C19	121.1 (2)
C7—C10—H10B	109.5	C23—C24—H24	119.5
H10A—C10—H10B	109.5	C19—C24—H24	119.5
C7—C10—H10C	109.5	C30—C25—C26	118.0 (2)
H10A—C10—H10C	109.5	C30—C25—C22	121.5 (2)
H10B—C10—H10C	109.5	C26—C25—C22	120.6 (2)
C12—C11—C14	107.5 (2)	C27—C26—C25	121.2 (3)
C12—C11—C6	110.4 (2)	C27—C26—H26	119.4
C14—C11—C6	111.31 (19)	C25—C26—H26	119.4
C12—C11—C13	110.4 (2)	C28—C27—C26	119.6 (3)
C14—C11—C13	106.5 (2)	C28—C27—H27	120.2
C6—C11—C13	110.45 (19)	C26—C27—H27	120.2
C11—C12—H12A	109.5	C29—C28—C27	120.3 (3)
C11—C12—H12B	109.5	C29—C28—H28	119.8
H12A—C12—H12B	109.5	C27—C28—H28	119.8
C11—C12—H12C	109.5	C28—C29—C30	120.6 (3)
H12A—C12—H12C	109.5	C28—C29—H29	119.7
H12B—C12—H12C	109.5	C30—C29—H29	119.7
C11—C13—H13A	109.5	C25—C30—C29	120.3 (3)
C11—C13—H13B	109.5	C25—C30—H30	119.9
H13A—C13—H13B	109.5	C29—C30—H30	119.9

### Hydrogen-bond geometry (Å, °)

**Table:**

*D*—H···*A*	*D*—H	H···*A*	*D*···*A*	*D*—H···*A*
N1—H1···O2^i^	0.87 (1)	1.97 (1)	2.827 (2)	174 (2)

Symmetry codes: (i) −*x*+1, −*y*+1, −*z*+1.
